# Geographic variation in the delivery of high-value inpatient care

**DOI:** 10.1371/journal.pone.0213647

**Published:** 2019-03-25

**Authors:** John Romley, Erin Trish, Dana Goldman, Melinda Beeuwkes Buntin, Yulei He, Paul Ginsburg

**Affiliations:** 1 Price School of Public Policy, University of Southern California, Los Angeles, California, United States of America; 2 School of Pharmacy, University of Southern California, Los Angeles, California, United States of America; 3 Vanderbilt University, Nashville, Tennessee, United States of America; 4 University of Maryland University College, Adelphi, Maryland, United States of America; 5 Brookings Institution, Washington D.C., United States of America; University of Pennsylvania, UNITED STATES

## Abstract

**Objectives:**

To measure value in the delivery of inpatient care and to quantify its variation across U.S. regions.

**Data sources / Study setting:**

A random (20%) sample of 33,713 elderly fee-for-service Medicare beneficiaries treated in 2,232 hospitals for a heart attack in 2013.

**Study design:**

We estimate a production function for inpatient care, defining output as stays with favorable patient outcomes in terms of survival and readmission. The regression model includes hospital inputs measured by treatment costs, as well as patient characteristics. Region-level effects in the production function are used to estimate the productivity and value of the care delivered by hospitals within regions.

**Data collection / Extraction methods:**

Medicare claims and enrollment files, linked to the Dartmouth Atlas of Health Care and Inpatient Prospective Payment System Impact Files.

**Principal findings:**

Hospitals in the hospital referral region at the 90^th^ percentile of the value distribution delivered 54% more high-quality stays than hospitals at the 10^th^ percentile could have delivered, after adjusting for treatment costs and patient severity.

**Conclusions:**

Variation in the delivery of high-value inpatient care points to opportunities for better quality and lower costs.

## Introduction

The Institute of Medicine has taken the position that “the only sensible way to restrain costs is to enhance the value of the health care system.”[1] Value is an elusive term in health care, but good value tends to mean high quality in relation to cost [2], and an array of initiatives in the private and public sectors seek to improve quality while containing costs. For example, the Centers for Medicare and Medicaid Services implemented its Hospital-Value Based Purchasing and Hospital Readmissions Reduction Programs in 2013, and has recently been rolling out Advanced Alternative Payment Models.[3]

This growing emphasis on value has outpaced the development of practical metrics of value performance.[4] For therapeutic drugs, cost-effectiveness has long been the standard to evaluate treatments. Nevertheless, a very lively dialogue about the appropriate framework for assessing value in pharmaceuticals has re-emerged. The measurement of value is still more unsettled in other settings, such as hospital care, even as reimbursement has been tied to indicators of quality and cost.

There are many reasons to suspect important variation in the value of care that is delivered. As scholars at the Dartmouth Institute first discovered and others have since confirmed, health care utilization and spending vary markedly throughout the U.S. Quality of care is also highly variable [5–7]; for example, among Medicare beneficiaries undergoing surgery in hospitals in 2009–2010, the 30-day risk-adjusted readmission rate was more than seventy percent higher at the 75^th^ percentile of its distribution than at the 25^th^ percentile.[8]

Such variability in both quality and cost—the core elements of value—is strongly suggestive of similar variation in value. Yet information about quality and cost is not directly informative about value in care delivery. If hospitals in one region have better quality but higher cost than those in another region, the formers’ care can be higher or lower-value than the latters’. If quality were higher but costs were the same, one could reach the qualitative conclusion that value is higher, but not the *quantitative* conclusion as to how much higher.

This study uses a production function framework to develop a value metric for inpatient care. Focusing on a high-prevalence medical condition -- heart attacks -- we assessed the value of the care delivered to Medicare beneficiaries hospitalized in 2013, and examined how value varies across regions.

## Methods

Providers deliver high-value care by producing good quality in relation to their costs.[2] Accordingly, we specify and analyze a production function for inpatient care; the output and inputs of our production function are detailed below. This analytical framework, and the closely related framework for cost functions, have been applied extensively to hospitals.[9–23]

The primary data source for our analysis was the Medicare Inpatient File from 2013. The medical claims in this file report patient diagnoses and procedures, demographic characteristics, charges and payments, dates of service, and the identity of the short-stay hospital. The research-identifiable 20 percent sample file that we used also reports patient ZIP codes. Where necessary, multiple claims were “rolled up” into a hospital stay.

We identified heart attack patients according to ICD-9 codes used in the Inpatient Quality Indicator (IQI) for risk-adjusted mortality from the Agency for Healthcare Research and Quality (AHRQ).[24] We then applied a number of additional criteria to create our final heart attack cohort. For example, patients who were transferred to other hospitals were excluded; complete criteria are shown in an appendix. We further limited the cohort in this study to elderly fee-for-service beneficiaries.[23]

To analyze the delivery of hospital care, we must define the output produced and the inputs used to produce it. We defined output to include not only quantity—as is common in studies of production—but also quality.[25] Specifically, following prior work [23], we measured the total number of “high-quality” stays in which the patient survived at least 30 days beyond the admission, and avoided an unplanned readmission within 30 days of discharge. Death dates were available from the Medicare Beneficiary Summary File; unplanned readmissions were identified based on the algorithm used by CMS for reporting and payment purposes.[26] These favorable outcomes are publicly reported and incorporated into current Medicare reimbursement; for example, mortality has been included in CMS’s Hospital Value-Based Purchasing Program since its introduction in fiscal year 2013.[27] Under our approach, only high-quality stays count toward the output that hospitals produce.

In a supplemental analysis, we also accounted for patient experience, multiplying the number of survivors without a readmission by the percentage of survey respondents who would have definitely recommended a hospital to friends and family from the Hospital Consumer Assessment of Healthcare Providers and Systems (HCAHPS).[28]

Our output measure makes an assumption about the tradeoff between the quantity and quality of hospital stays. In particular, output is unchanged if quality increases by one percent while the quantity of stays decreases by one percent. To assess the robustness of our findings to this assumption, we performed a sensitivity analysis that used the number of stays (regardless of outcomes) as the dependent variable, and included mortality, readmission and satisfaction rates as explanatory variables in the production model. In addition to health care, hospitals produce graduate medical education, and so all models included variables for residents-per-bed thresholds used in the literature and reported in the Inpatient Prospective Payment System (PPS) Impact File.[29–32] To address the provision of tertiary care, all analyses also included indicator variables for delivery of advanced cardiac and neurological procedures, as defined in the Dartmouth Atlas of Health Care.[33]

To characterize hospital inputs—the key explanatory variable in the production model -- we followed the literature on inpatient care in using an aggregate measure.[5, 6, 20, 21, 34] Specifically, we measured the total cost to each hospital of treating patients in the heart attack cohort (including patients with unfavorable outcomes.) To do so, we first converted total hospital charges covered by Medicare to costs based on the cost-to-charge ratios submitted by hospitals to CMS as part of their cost accounting reports, which are reported in the CMS Impact File. We then adjusted for geographic differences in labor prices using the hospital wage index, also from the Impact File; this adjustment was applied to the labor-related portion of the base PPS payment rate. We measured costs in 2014 US dollars, based on the medical component of the consumer price index. In a sensitivity analysis, we did not adjust for area wages; this analysis assessed the impact of wage adjustment, as there have been concerns about mismeasurement of wages.[35]

We followed prior work in addressing patient severity.[5, 6, 23, 36] For each hospital, we included variables for the proportions of patients with heart attacks in specific locations based on diagnosis codes (for example, 410.2 for acute myocardial infarction of the inferolateral wall.)[21] We also included the proportions of a hospital’s patients with different numbers of Charlson co-morbidities in the medical claims for heart attack stays [37], as well as the average socio-demographic characteristics of patients’ zip codes from the 2009–2013 American Community Survey (for example, the poverty rate and the percentage of elderly residents with disabilities.)[38] To further address patient severity, we adjusted for the likelihood of death during the hospital stay, using the risk adjustment model developed by clinical experts as an input into AHRQ’s heart attack mortality IQI.[24] The AHRQ risk model predicts the probability that a patient dies based on her age and sex, transfer from another hospital, and All Payer Refined-Diagnosis Related Group (APR-DRG); each APR-DRG includes its own mortality-risk scale.[39] We included covariates for average age and proportion female, which could be related to treatment costs as well as patient severity. We also adjusted for race and ethnicity. In a sensitivity analysis, we excluded all diagnosis-based covariates while adding the proportion of patients admitted from the emergency room or transferred from another hospital, because there is some evidence of regional differences in how conditions are diagnosed.[40, 41]

In studying inpatient treatment of heart attack in 2013, we focus on the value of care delivered within areas defined by hospital referral regions (HRRs) from the Dartmouth Atlas of Health Care.[33] Thus, a high-value HRR is one whose hospitals tended to produce more stays -- or a better rate of high-quality stays than expected -- given its levels of treatment costs and patient severity. We implemented our model by assuming that HRR-level value was normally distributed and applying the method of maximum likelihood.[42, 43] For representativeness, each hospital-level observation was weighted by the number of patients treated. Our approach produced an estimate of the proportion of (unmeasured) variation in output resulting from differences between HRRs in the average performance of their hospitals, compared to the differences around the average among the hospitals within the HRRs (this latter variation reflects hospital-level value as well as randomness.) This approach did, however, make the assumption that value was systematically unrelated to other factors, such as patient severity across areas. In a sensitivity analysis, we relaxed this assumption using fixed-effects regression to assess HRRs.

These analyses produced estimates of value for each HRR, adjusted for the reliability of the value performance signal based on the size of the area. We transformed these HRR-specific estimates into a value index with a national mean of 100.

We explored the relationship between quality, cost and value. While our production framework analyzed total costs in relation to the total number of high-quality stays, it is natural and commonplace to assess provider cost and quality based on *average* performance. We therefore compared our value index to cost per stay and the rate of high-quality stays, adjusting each for the patient and hospital characteristics noted above in independent regressions. The appendix provides further information on the data and analyses, including additional robustness checks.

## Results

In our 2013 sample, 33,713 elderly fee-for-service beneficiaries were admitted with a heart attack to 2,232 hospitals in 304 hospital referral regions (HRRs) with at least 11 heart-attack stays in our database of Medicare claims. Fifty-one percent of these patients were female, and the average age was 80 years. The cost of these hospital stays averaged $14,900 in 2014 dollars. In terms of outcomes, 87% of patients survived at least 30 days beyond the admission, while 86% of these survivors avoided an unplanned readmission within 30 days of discharge. The overall rate of high-quality hospital stays (survival without readmission) was 74%.

**Fig 1:**
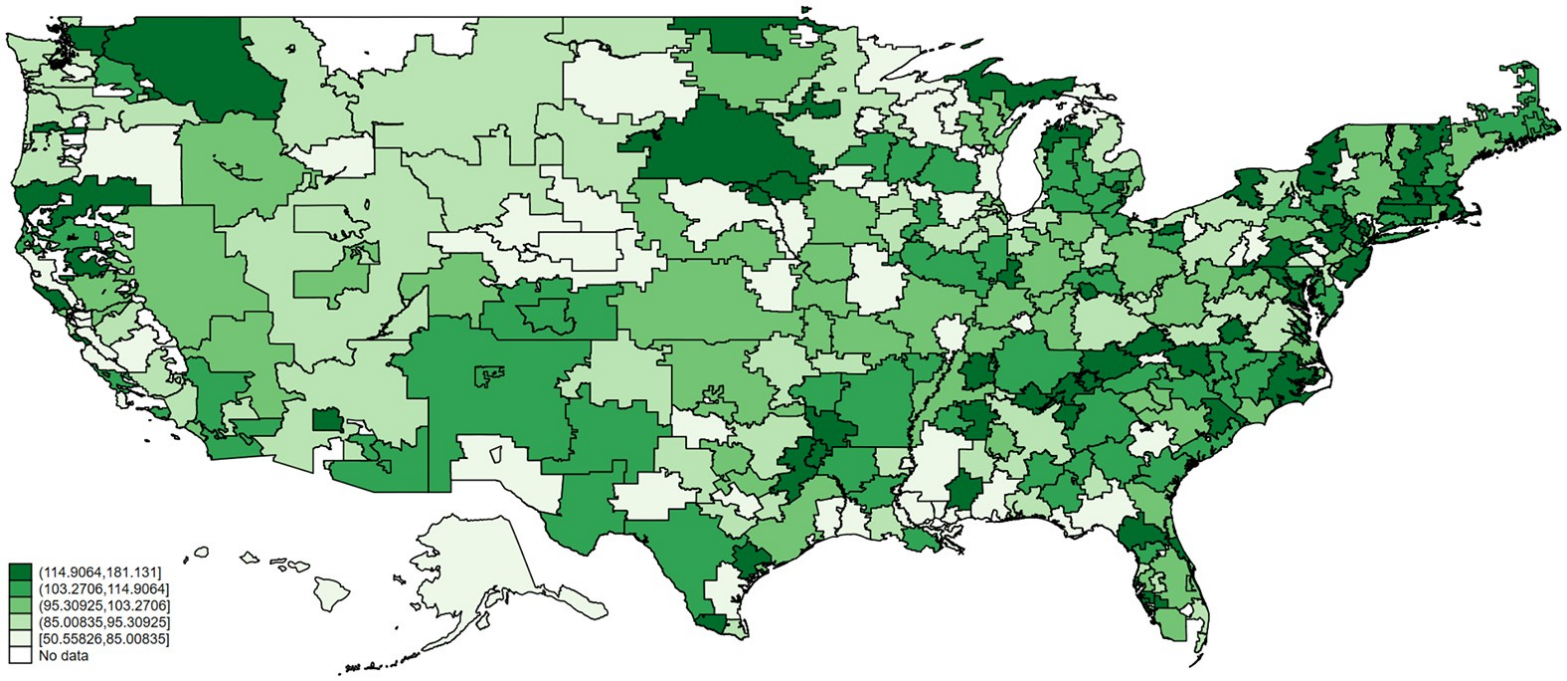
Value index for inpatient heart attack care in 2013, by hospital referral region grouped into quintiles. Note: Darker green indicates higher value.

Based on quality of care, treatment cost, patient severity and hospital characteristics (including teaching status), our analytic framework quantifies value in inpatient heart attack care across the U.S. The national map in Fig 1 shows the value of care delivered in each HRR, with dark green indicating the highest quintile of value. Compared to the U.S. average of 100, Miami’s score on our value index was 87. Thus, hospitals in Miami produced 13% *fewer* high-quality hospital stays (87%—100% = -13%) than hospitals in the average U.S. region would have been expected to produce if their costs and patients had been the same. As another example, Everett, Washington performed better than the national average, with a value index score of 122. Both of these scores were statistically distinguishable (with 95% confidence) from the national average; among all HRRs, 71% were significantly different from 100.

**Fig 2:**
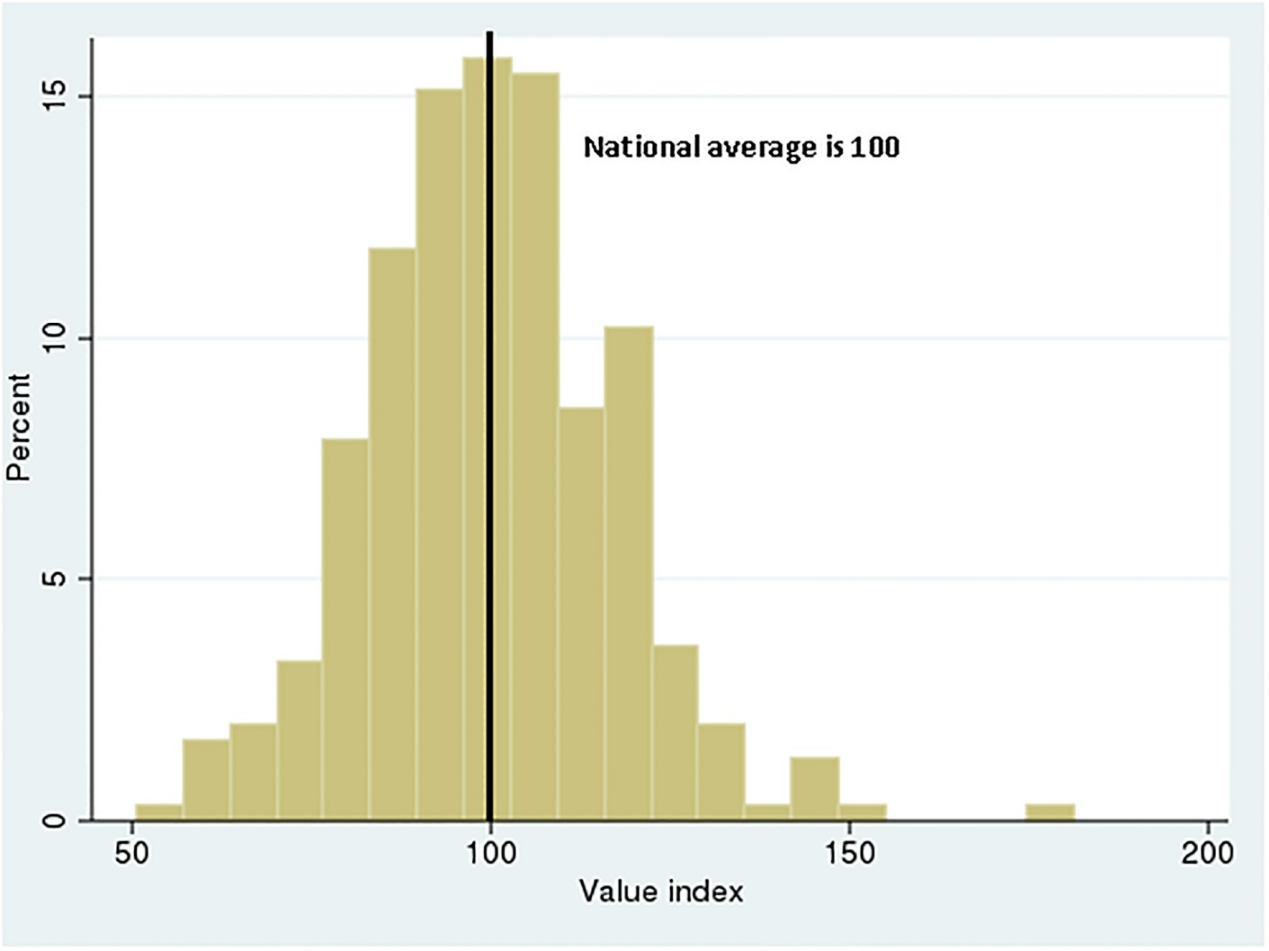
Distribution of inpatient heart attack care value index across hospital referral regions.

The range of value index scores is shown in the histogram in Fig 2. About one in 8 U.S. regions had a value index in excess of 120, thus delivering at least 20% more value than the national average, that is, 20% more high-quality heart attack stays than the average region after adjusting for treatment cost and patient severity. The value index for the HRR at the 90^th^ percentile of the distribution, compared to the score at the 10^th^ percentile, exhibited a ratio of 1.54:1. That is, value in care delivery was 54% higher for the region whose performance exceeded 9 out of 10 of all regions, compared to the region whose performance exceeded only 1 out of 10 regions. For the components of value, adjusted costs and quality of care, the corresponding 90–10 ratios were 1.42:1 and 1.36:1, respectively. In terms of value in care delivery, hospitals in the *median* HRR would have to increase their performance by 22% to reach the top decile (i.e., the 90–50 ratio was 1.22.) These differences between HRRs accounted for 32% of the unmeasured variation in hospital output.

**Fig 3:**
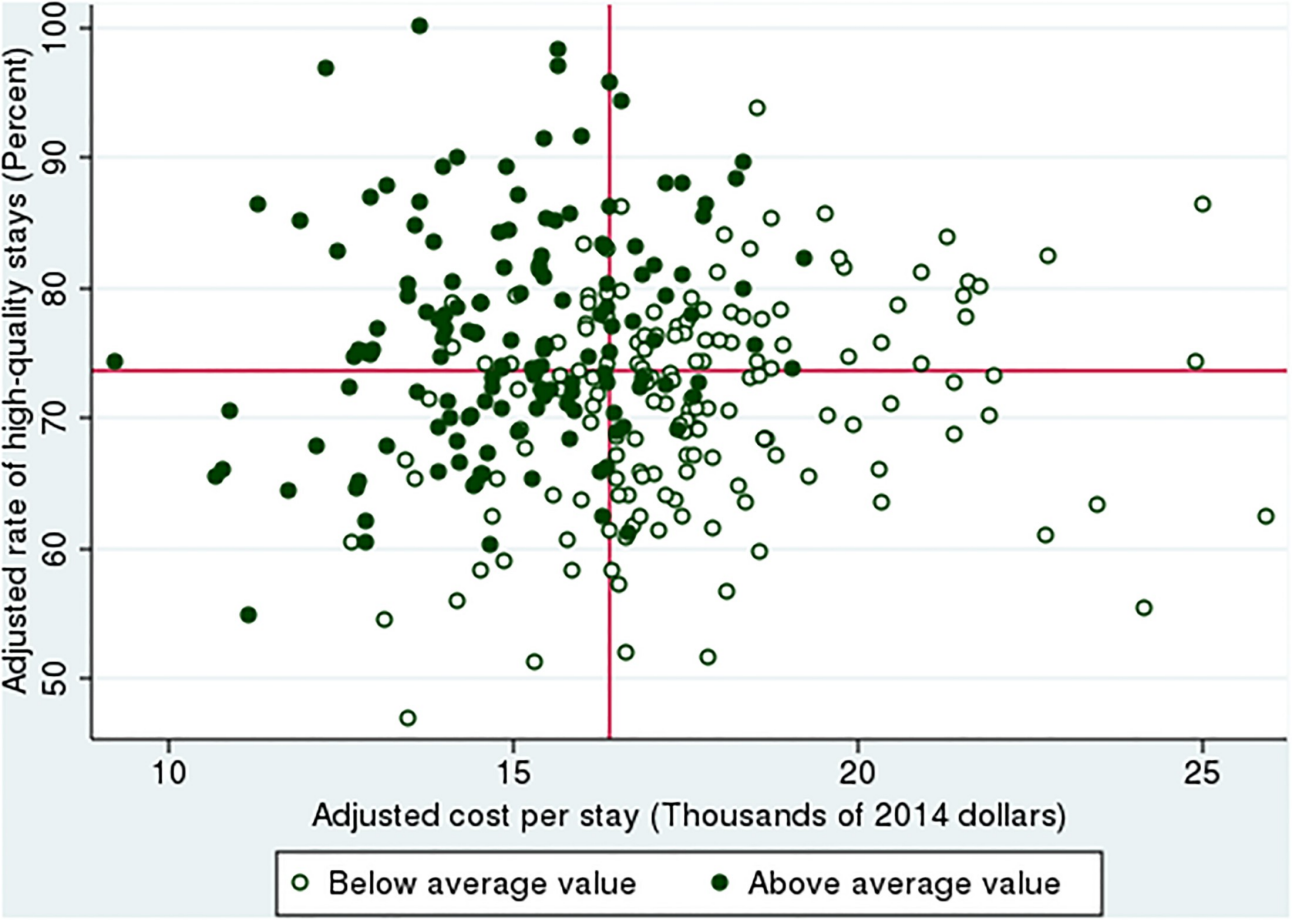
Quality, cost and value in the delivery of inpatient heart attack care among hospital referral regions. Notes: Red lines indicate national averages. High-quality stays is defined by 30-day survival without an unplanned readmission and willing to recommend hospital. Quality and cost are adjusted as explained in manuscript.

Fig 3 shows quality, cost and value in the delivery of inpatient care for heart attack. Specifically, HRRs are characterized as above- or below-average in value, and are located within quadrants defined by average cost and quality. In the upper left quadrant, adjusted cost is below the national average, while adjusted quality is above average. Within this quadrant, 78% of HRRs were above-average in value, with value index scores exceeding 100. In the bottom right quadrant, cost is above average and quality below average. Here only 13% of HRRs are above average in value. When costs *and* quality are above average—the upper right quadrant—31% of regions deliver above average value, with higher quality than would have been expected given the high costs. When both costs and quality are below average -- the bottom left quadrant—some HRRs (specifically, 63%) are also above average. Among all regions with above-average value, 55% were below average in terms of adjusted quality or above average in cost. The regression results (reported in the appendix) imply that a region with 10% higher cost than another region lies on the same production function -- and thus delivers equivalent value -- if the higher-cost region also delivers 8% more quality (in terms of the rate of high-quality stays).

In a sensitivity analysis, we relaxed the assumption that HRR-level value was independent of factors such as patient severity. The resulting (“fixed effects”) value index scores for HRRs were quite similar to the scores from the primary analysis (ρ = +0.789, *p* < 0.001.) In another sensitivity analysis, we redefined the dependent variable of hospital output as the number of heart attack stays and included the rates of 30-day survival and unplanned readmission as regression covariates, and again found similar value index scores for HRRs (ρ = +0.848, *p* < 0.001.) The value index scores were also similar when we incorporated the patient experience into hospital output (ρ = +0.942, *p* < 0.001.) Finally, we found that the value scores were not highly sensitive to the adjustment of costs for area wages or to the measurement of patient severity based on recorded diagnoses; for both of these sensitivity analysis, the correlation coefficient with the results of our primary analysis exceeded +0.90. The scores were also insensitive to a number of other robustness tests described in the appendix.

## Discussion

This study has used a framework for the production of high-quality health care to develop and implement a measure of the value of inpatient care among Medicare beneficiaries with heart attacks in 2013. Defining high-quality hospital stays by survival at least thirty days beyond the admission and avoidance of an unplanned readmission within thirty days of discharge, we documented substantial variation in the value of the care that was delivered. In a key finding, hospitals located in the hospital referral region (HRR) at the 90^th^ percentile of the value distribution delivered 54% more high-quality stays than hospitals located in the HRR at the 10^th^ percentile would have been expected to produce if their treatment costs and patient severity had been the same. Our findings were robust to a number of alternative approaches to value measurement.

This variability in value is of a similar magnitude to, but somewhat larger than, the variability in regional per capita Medicare *spending* found in the recent study by the National Academy of Medicine (NAM) of geographic variation in U.S. health care (there the adjusted 90:10 ratio for HRRs was 1.42.)[44] The NAM study also found substantial variation in Medicare spending within a region. In our analysis, differences between HRRs accounted for only a third of unobserved variation in the production of high-quality inpatient care for heart attacks. While some of the remaining variation resulted from randomness in the production process, the scale of the sub-regional variation suggests that individual hospitals within HRRs differed in the value of the care that was delivered.

This variability in the value of care points to potential opportunities for higher quality, lower costs, or both within the health care system. The prospect of such performance improvements has spurred efforts by system participants and policy makers to experiment with new models of delivery and financing. For example, in the context of inpatient care, the Centers for Medicare and Medicaid Services (CMS) implemented a Hospital Value-Based Purchasing Program in fiscal year 2013, in an effort to incentivize value in care delivery.[27, 45] Initially, the program operationalized value solely on the basis of quality-of-care metrics. CMS later proposed that a cost measure—namely, Medicare Spending per Beneficiary (MSPB)—be incorporated into value-based purchasing, starting in fiscal year 2017. The proposed 2017 rule for the inpatient prospective payment system places a weight of twenty-five percent on the “Efficiency and Cost Reduction” domain assessed according to the MSPB measure, with the remaining weight distributed among a number of quality-oriented domains.[46]

This kind of policy choice effectively takes a stance on the nature of the relationship between cost and quality in the delivery of high-value care. Our own framework for value assessment provides empirical evidence about the tradeoff between cost and quality. In particular, for inpatient heart attack care, a ten-percent decrease in treatment costs would be consistent with greater value if the rate of “high-quality” stays—achieving 30 day survival without an unplanned readmission -- did not decrease by more than 8 percent. While other considerations are certainly relevant (for example, basing payment partly on improvement so as to incentivize and reward poor performers), value-oriented policy should be informed by the real-world relationship among quality, cost and value.

In our findings, it is noteworthy that hospitals in some areas with high risk-adjusted costs nevertheless tended to deliver good value, because their adjusted quality of care was also high. Conversely, some areas with relatively low quality were high-value, due to sufficiently low costs. The Medicare Payment Advisory Commission (MedPAC) deems hospitals to be efficient if their risk-adjusted quality of care exceeds a uniform threshold, while their costs fall below some threshold.[47] This kind of approach can reliably identify high-value providers, achieving good specificity—that is, turning up few false positives—in the language of applied statistics. In our study, roughly nine out of ten HRRs with above-average quality and below-average cost delivered above-average value. Yet this approach can miss instances of favorable performance (thus lacking sensitivity, resulting in false negatives.) In our study, half of regions with above-average value were below-average with respect to quality, or above-average with respect to cost. To be sure, our value measure includes statistical noise—even if our production model does not introduce any systematic biases—yet it contrasts with the MedPAC approach in recognizing the tradeoff between quality and cost.

This study does have a number of limitations. To begin with, our conclusions may not be generalizable beyond the condition studied. Nevertheless, heart attack is an important contributor to hospital admissions in the United States, accounting for 3.7 million stays in 2010.[48] Diseases of the heart accounted for almost 600,000 deaths in that year.[49]

Another limitation is that in operationalizing hospital output, we measured quality of care based on mortality, unplanned readmissions and patient satisfaction. While these outcomes are of considerable salience to patients and policy makers, other outcomes (such as functional status and quality of life after hospitalization) may also merit attention in analyses of the value of inpatient treatment. In addition, patient outcomes reflect not only the care received while in the hospital, but also a range of other factors (for example, post-acute care and personal behavior). In measuring patient satisfaction, the HCAHPS survey was not specific to heart attack stays nor to Medicare patients, though reported measures are adjusted for factors including age and service line, and CMS uses HCAHPS to evaluate cardiac care.[50]

Finally, our measure of hospital inputs did not capture the cost of services provided by physicians who were not hospital employees. Incorporating other kinds of health care costs represents a fruitful direction for future research.

Despite these limitations, this study strongly suggests that there is wide variation across the United States in the value of the inpatient care delivered to Medicare beneficiaries, just as there are substantial differences in quality and cost of care.[1] Variation in value points to the existence of important opportunities for achieving uniformly high-value care, with better quality and / or lower costs for Medicare beneficiaries who suffer heart attacks. There are likely lessons to be learned from the practices and cultures of high-performing hospitals, to then be implemented by providers whose value in care delivery lags behind. In the context of quality of care, researchers and practitioners have explored the factors that distinguish hospitals with superior patient outcomes.[51, 52] A necessary first step in any such undertaking is measurement, in our case, of value in care delivery. Moreover, to enhance provider performance and effective policymaking, it is critically important to understand value in broader settings, outside of heart attack, at sites of care other than hospitals, and indeed in the delivery of both episodes of care and population health across multiple providers and within health systems.

## Supporting information

S1 File Appendix. (DOCX)
