# Supplementary material for: Geographic variation in the delivery of high-value inpatient care
Source: PLoS One. 2019 Mar 25;14(3):e0213647. doi: 10.1371/journal.pone.0213647 (PMC6433342; doi:10.1371/journal.pone.0213647)
Supplement: S1 File — (DOCX) [file pone.0213647.s001.docx]

**Appendix**

We analyzed the following production function for U.S. hospitals:

| $\ln Y_{h}= \beta_{0}+\beta_{I}\ln I_{h}+\boldsymbol{\beta}_{\boldsymbol{X}}\boldsymbol{X}_{\boldsymbol{h}}+\nu_{r}+\epsilon_{h}\text{,}$ | (A1) |
| --- | --- |

in which ln *Y_h_* was the natural logarithm of the output produced by hospital *h*, *I_h_* was a measure of production inputs used by the hospital (also in logarithms), ***X_h_*** was a group of control variables for patient severity and hospital characteristics (described below), *ν_r_* was a region-specific factor which was common to hospitals within a hospital referral region (HRR), and *ε_h_* was a random factor specific to the hospital.

*Analysis sample*

We analyzed the delivery of inpatient care to elderly fee-for-service Medicare beneficiaries admitted to a short-term acute care hospital in the 50 states and D.C. with a principal diagnosis of acute myocardial infarction (AMI, or heart attack) in calendar year 2013.

Our primary source of data was a 20% random sample (with respect to beneficiaries) of the research-identifiable version of the Medicare Inpatient File.([1](#_ENREF_1)) Where necessary, we combined inpatient claims to create a stay-level data set equivalent to the Medicare Provider Analysis and Review (MedPAR) File.([2](#_ENREF_2))

AMI stays were identified according to the selection criteria specified for Inpatient Quality Indicator (IQI) #15 from the Agency for Healthcare Research and Quality (AHRQ), version 5.0.([3-5](#_ENREF_3)) These criteria are shown in Appendix Exhibit 1. We restricted the sample to beneficiaries who were 65 years old or older on the date of admission. A beneficiary was fee-for-service if the HMO indicator variable from the Base (A/B/D) segment of the Beneficiary Summary File was equal to zero in the month of admission.([6](#_ENREF_6), [7](#_ENREF_7))

Hospital stays in the 50 states and D.C. were identified by the first two digits of the Medicare provider number. Stays at short-term acute care hospitals were identified based on the last four digits of the provider number, consistent with the algorithm that defines the short-stay indicator variable in MedPAR.([8](#_ENREF_8))

*Specification of production function*

We measured a hospital’s output $Y_{h}$ by the number of AMI patients who survived at least 30 days beyond the admission, avoided an unplanned discharge within 30 days of discharge, and would have definitely recommended the hospital to friends and family. We measured the number of surviving patients based on the verified date of death variable in our stay-level data set.

The rate of unplanned readmissions was measured within our AMI cohort according to the algorithm used by the Centers for Medicare and Medicaid Services (CMS) for reporting and payment purposes; notably, the selection criteria for the cohort overlap with those the AHRQ IQI. We used the 2014 version of the unplanned readmissions algorithm which is characterized in Appendix Exhibits 2 and 3.([9](#_ENREF_9), [10](#_ENREF_10)) We did not impose the 12 month “look back” on Medicare claims, because this data is needed only to produce CMS’s risk-adjusted measure; in our analysis, readmission was risk-adjusted based on the patient severity measures in ***X_h_***. We used our stay-level data set to calculate the rate of avoidance of unplanned readmissions among AMI patients admitted during 2013 and discharged at least 30 days before January 1, 2014, and multiplied the number of survivors by this rate.

To finalize our output measure, we multiplied the number of survivors without an unplanned readmission by the percentage of patients who would definitely recommend the hospital to friends and family. This percentage was obtained from patient surveys collected during 2013 through the Hospital Consumer Assessment of Healthcare Providers and Systems.([11](#_ENREF_11), [12](#_ENREF_12))

Turning to production inputs $I_{h}$, we measured the hospital’s total cost of treating all AMI patients (including decedents, etc.) To do so, we first calculated total charges from the inpatient (Part A) claims. We then converted charges to costs using cost-to-charge ratios from the Impact File for the Acute Inpatient Prospective Payment System. Finally, we adjusted costs for differences across areas in labor markets. Specifically, we divided the labor portion of total costs by the Hospital Wage Index, obtained from the Impact File; the labor portion was calculated by multiplying total costs by the national labor share in 2013 (69.6%).([13-15](#_ENREF_13)) To deal with the reporting lag in the Impact Files, we used the file corresponding to fiscal year 2016, based on personal conversation with CMS staff.

Turning to patient severity, we included a range of measures in ***X_h_***. We used predicted inpatient mortality risk, obtained by applying the risk adjustment model for AHRQ’s AMI IQI to our stay-level data set; this risk adjustment model is shown in Appendix Exhibit 4.([16](#_ENREF_16)) We also used diagnosis codes in the stay-level data set to characterize the location of the AMI within the heart and to identify comorbidities. In addition, demographic characteristics from the data set were used. Finally, we included the average value of sociodemographic characteristics from a hospital’s patients’ zip codes, as reported in our stay-level data set. Specifically, we used all matches between zip codes and Zip Code Tabulation Areas from the 2009-2013 American Community Survey.([17](#_ENREF_17))

Turning to hospital characteristics, we included teaching status and the provision of advanced inpatient services in ***X_h_***. To measure teaching status, we included an indicator variable for the presence of any medical residents, and another indicator variable for more than 0.6 residents per bed, as reported in the Impact File. For advanced services, we created indicator variables for at least two admissions in 2013 within our 20% sample with specific diagnostic related group (DRG) codes for major cardiovascular procedures and neurosurgery, updated from the original Dartmouth Atlas of Health Care.^[[1]](#footnote-2)^([18](#_ENREF_18), [19](#_ENREF_19))

Summary statistics for the analysis sample are shown in Appendix Exhibit 5.

*Estimation of Value in Care Delivery*

We estimated the production function in equation (A1) using the **mixed** command in Stata, version 14.1.([20](#_ENREF_20), [21](#_ENREF_21)) The hospital-level observations were weighted by the number of admissions for representativeness. The estimation model included HRR-level random effects, assumed to be distributed normally; each hospital’s HRR was obtained from a Dartmouth Atlas of Health Care database that included Medicare provider numbers.([6](#_ENREF_6), [22](#_ENREF_22)) The model was estimated by the method of maximum likelihood, starting with the expected-maximization algorithm. The estimation results from the primary analysis are shown in Appendix Exhibit 6.

Post-estimation, the **mixed** command produced best linear unbiased predictors (BLUPs) of the HRR random effects, denoted as $\hat{\nu}_{r}$. The command also produced BLUP standard errors, which were used to test for statistically significant differences from the sample mean (by construction) of zero.

Because hospital output was measured in logarithms, the BLUPs were logarithmically scaled. For greater interpretability, we transformed them into a measure of value in care delivery, according to the following formula:

$V_{r}=100\times\frac{exp\left( \hat{v}_{r} \right)}{{\sum_{r´} exp\left( \hat{v}_{r´} \right)}/{n_{r}}}$, (A2)

in which $n_{r}$ is the number of HRRs, and the resulting measure has a mean of 100 by construction. The measure can also be interpreted as average hospital productivity within a region, because provider productivity generates value in care delivery.([23](#_ENREF_23))

*Sensitivity analyses*

We performed a series of sensitivity analyses, and now describe each. The first four are discussed in the manuscript.

1. Fixed effects versus random effects: We used Stata’s **xtreg** command to produce fixed-effects estimates of $v_{r}$. The correlation between the sets of area-level value index scores was +0.789.

2. Alternative specification of output in terms of quantity and quality: We redefined $Y_{h}$ in equation (A1) to be the total number of hospital stays, and included the rates of survival and avoidance of readmission among survivors as model covariates. The correlation between the value scores was +0.848.

3. Risk adjustment not based on diagnoses: We excluded AHRQ predicted inpatient mortality, Charlson-Deyo comorbidities, and heart-attack location from equation (A1). We included the percentage of admissions whose sources were the emergency department and (independently) another short-term acute care hospital (stays which resulted in transfer to another hospital were already excluded according to the IQI selection criteria.) Admissions from the emergency department were identified by positive charges for emergency room services, derived from revenue centers in the inpatient claims according to the algorithm used in MedPAR.([24](#_ENREF_24)) A stay was a transfer from another hospital if the source of admission variable (originally from the Inpatient File) was not missing and equal to 4. The correlation between the value scores was +0.942.

4. Cost not adjusted for area wages: We did not adjust costs for area wages using the CMS Hospital Wage Index. The correlation between the value scores was +0.911.

5. Hospital-specific wage index: To measure cost, we replaced the area-level wage index with the hospital-specific index also reported by CMS. The correlation between the value scores was +0.983.

6. Operating costs only: We measured operating costs only using operating cost-to-charge ratios from CMS. The correlation between the value scores was +0.977.

7. Extreme cost-to-charge ratios: We winsorized extreme (operating plus capital) ratios at the 2.5^th^ and 97.5^th^ percentiles. The correlation between the value scores was +0.998.

8. Process quality: We added the most widely reported measure on Hospital Compare during 2013 as a covariate, specifically, proportion of AMI patients prescribed aspirin at discharge. The correlation between the value scores was +0.942.

9. Further risk adjustment: We added the logged proportions of 1) AMI patients who were also eligible for Medicaid, as measured using the Master Beneficiary Summary File, and 2) hospital days for Medicare beneficiaries who also received Supplemental Security Income in fiscal year 2013, as reported by CMS. The correlation between the value scores was +0.937.

*References*

1. Research Data Assistance Center. Inpatient RIF. Available from: <https://www.resdac.org/cms-data/files/ip-rif>.

2. Research Data Assistance Center. MedPAR RIF. Available from: <https://www.resdac.org/cms-data/files/medpar-rif>.

3. Agency for Healthcare Research and Quality. Inpatient Quality Indicators Overview. Available from: <http://www.qualityindicators.ahrq.gov/modules/iqi_overview.aspx>.

4. Agency for Healthcare Quality and Research. Guide to Inpatient Quality Indicators. 2008.

5. Agency for Healthcare Research and Quality. Inpatient Quality Indicators Technical Specifications - Version 5.0, March 2015. Available from: <http://www.qualityindicators.ahrq.gov/Archive/IQI_TechSpec_ICD9_v50.aspx>.

6. Dartmouth Institute. The Dartmouth Atlas of Health Care. Available from: <http://www.dartmouthatlas.org/>.

7. Research Data Assistance Center. Master Beneficiary Summary File. Available from: <https://www.resdac.org/cms-data/files/mbsf>.

8. Research Data Assistance Center. MEDPAR Short Stay/Long Stay/SNF Indicator Code. Available from: <https://www.resdac.org/cms-data/variables/MEDPAR-Short-StayLong-StaySNF-Indicator-Code>.

9. CMS.gov. Measure Methodology. Available from: <https://www.cms.gov/Medicare/Quality-Initiatives-Patient-Assessment-Instruments/HospitalQualityInits/Measure-Methodology.html>.

10. CMS Readmission Measures. 2014 Measure Calculation Package.

11. Data.Medicare.gov. Hospital Compare data archive. Available from: <https://data.medicare.gov/data/archives/hospital-compare>.

12. Data.Medicare.gov. HOSArchive_20141218.zip. Available from: <http://medicare.gov/download/HospitalCompare/2014/December/HOSArchive_20141218.zip>.

13. CMS.gov. FY 2016 IPPS Final Rule Home Page. Available from: <https://www.cms.gov/Medicare/Medicare-Fee-for-Service-Payment/AcuteInpatientPPS/FY2016-IPPS-Final-Rule-Home-Page.html>.

14. CMS.gov. FY 16 Impact File. Available from: <https://www.cms.gov/Medicare/Medicare-Fee-for-Service-Payment/AcuteInpatientPPS/Downloads/FY2016-CMS-1632-FR-Impact.zip>.

15. CMS.gov. CMS-1632-F and IFC, CMS-1632-CN2 and Changes due to The Consolidated Appropriations Act of 2016. Available from: <http://www.gpo.gov/fdsys/pkg/FR-2015-08-17/pdf/2015-19049.pdf>.

16. Agency for Healthcare Research and Quality. Inpatient Quality Indicators Parameter Estimates, Version 5.0. Available from: <http://www.qualityindicators.ahrq.gov/Downloads/Modules/IQI/V50/Parameter_Estimates_IQI_50.pdf.pdf>.

17. U.S. Census Bureau. American Community Survey. Available from: <https://www.census.gov/programs-surveys/acs/>.

18. Wennberg J, Cooper M. The Dartmouth atlas of health care. The Center for the Evaluative Clinical Sciences, Dartmouth Medical School, American Hospital Publishing. 1996:15-20.

19. CMS.gov. DRG Data Files. Available from: <https://www.cms.gov/Medicare/Medicare-Fee-for-Service-Payment/AcuteInpatientPPS/Downloads/FY_2008_FR_DRG_Files.zip>.

20. StataCorp. Stata. Available from: <http://www.stata.com/>.

21. StataCorp. Stata Multilevel Mixed-Effects Reference Manual, Version 13. Available from: <https://www.stata.com/manuals13/me.pdf>.

22. Dartmouth Institute. Hospital to HSA/HRR Crosswalk, 2007. Available from: <http://www.dartmouthatlas.org/downloads/geography/hosp_hsa_hrr_2007.xls>.

23. Romley JA, Goldman DP, Sood N. US hospitals experienced substantial productivity growth during 2002-11. Health Aff (Millwood). 2015;34(3):511-8.

24. Research Data Assistance Center. MEDPAR Emergency Room Charge Amount. Available from: <https://www.resdac.org/cms-data/variables/MEDPAR-Emergency-Room-Charge-Amount>.

**Appendix Exhibit 1: Selection Criteria for Acute Myocardial Infarction Admissions from AHRQ Inpatient Quality Indicator #15**


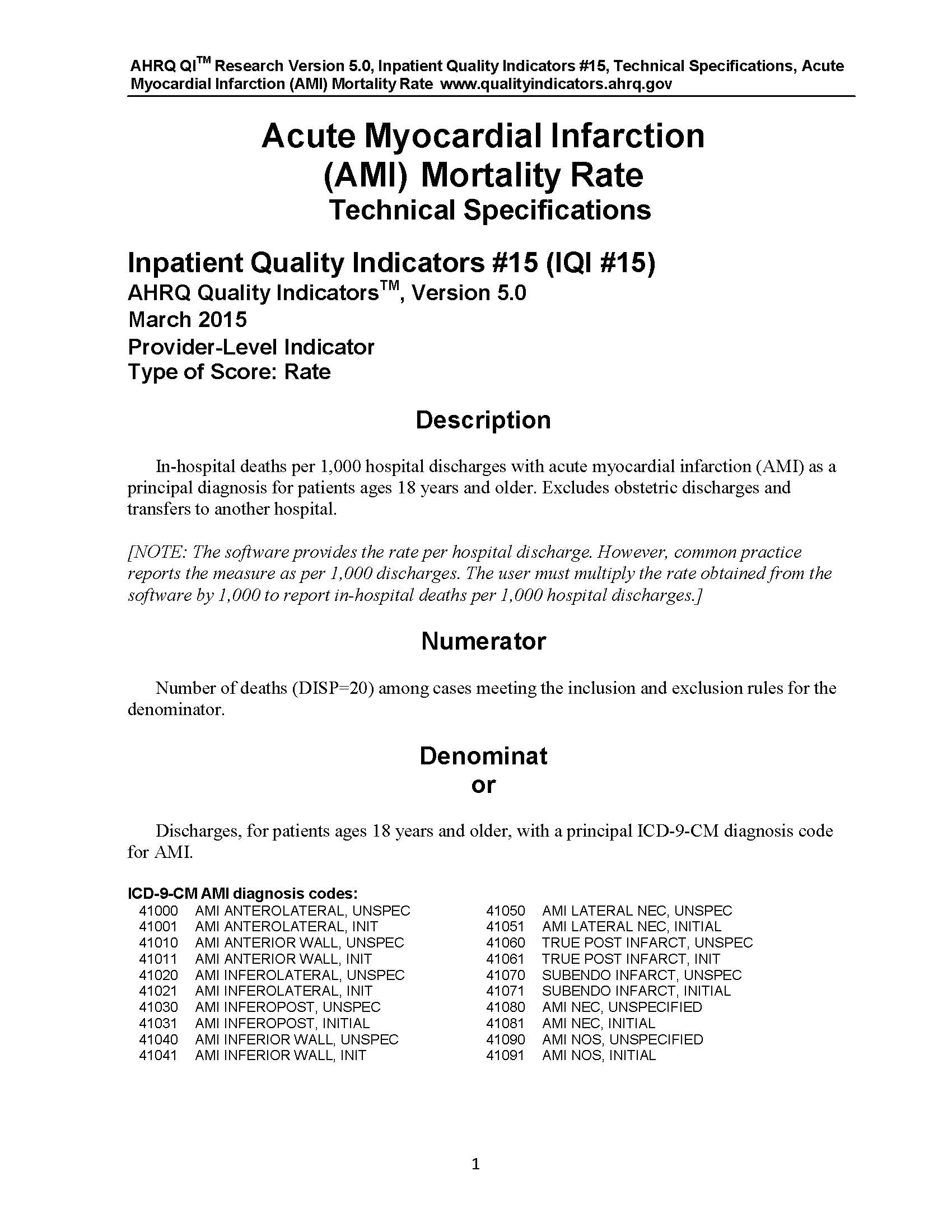


**Appendix Exhibit 1, Continued**


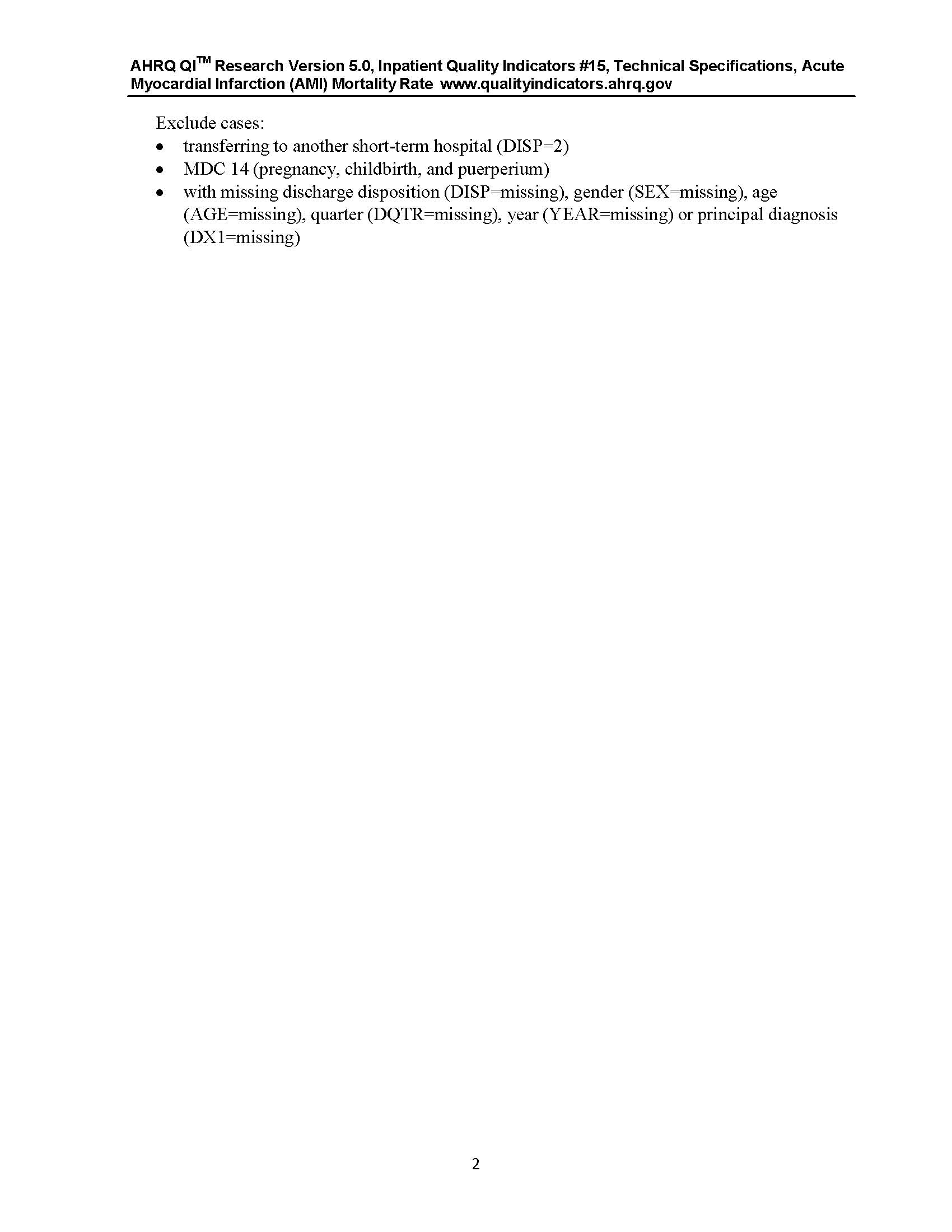


**Appendix Exhibit 2: Selection Criteria for**

**
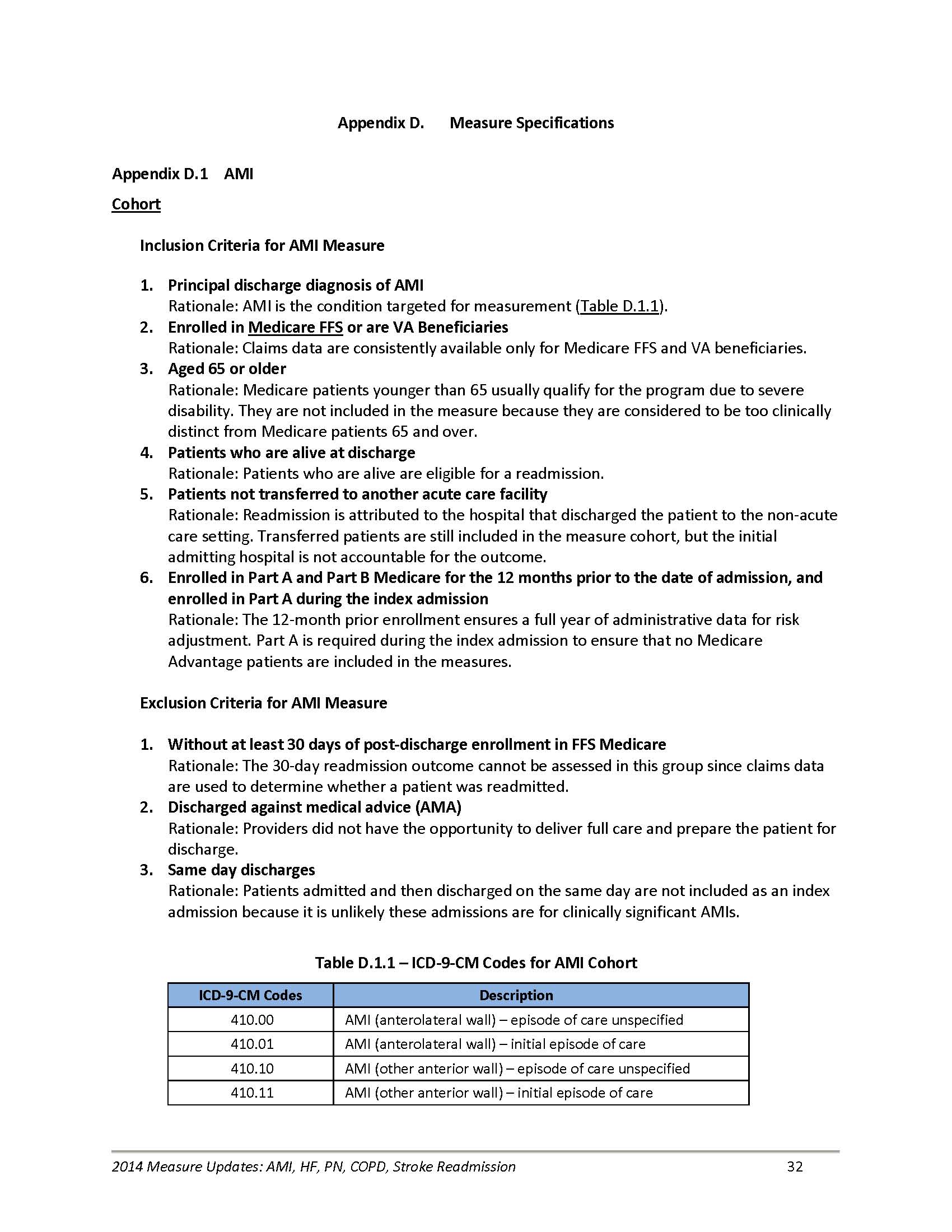
CMS AMI Readmission Measure**

**Appendix Exhibit 3: Details on Identification of**

**
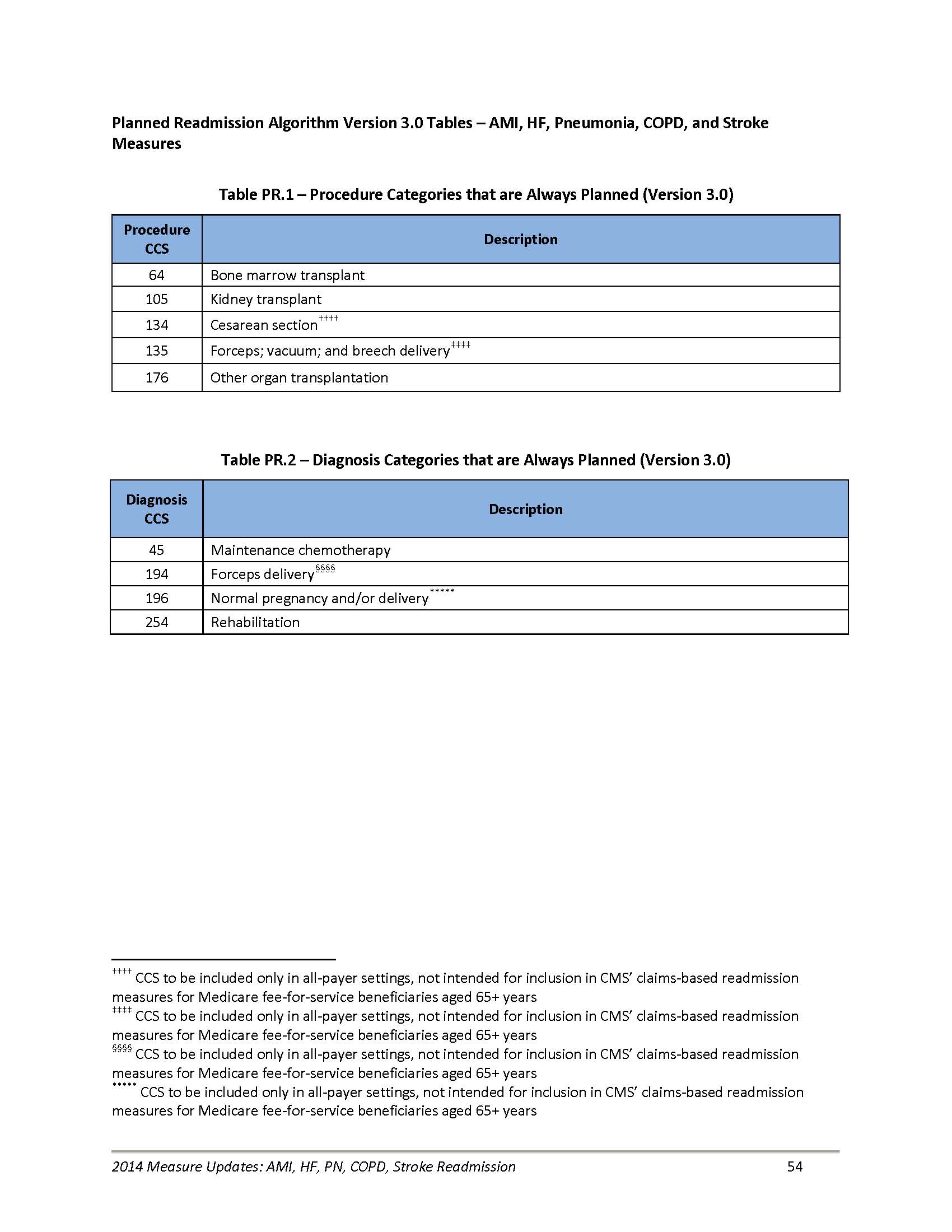
Unplanned Readmissions**

**Appendix Exhibit 3, Continued**

**
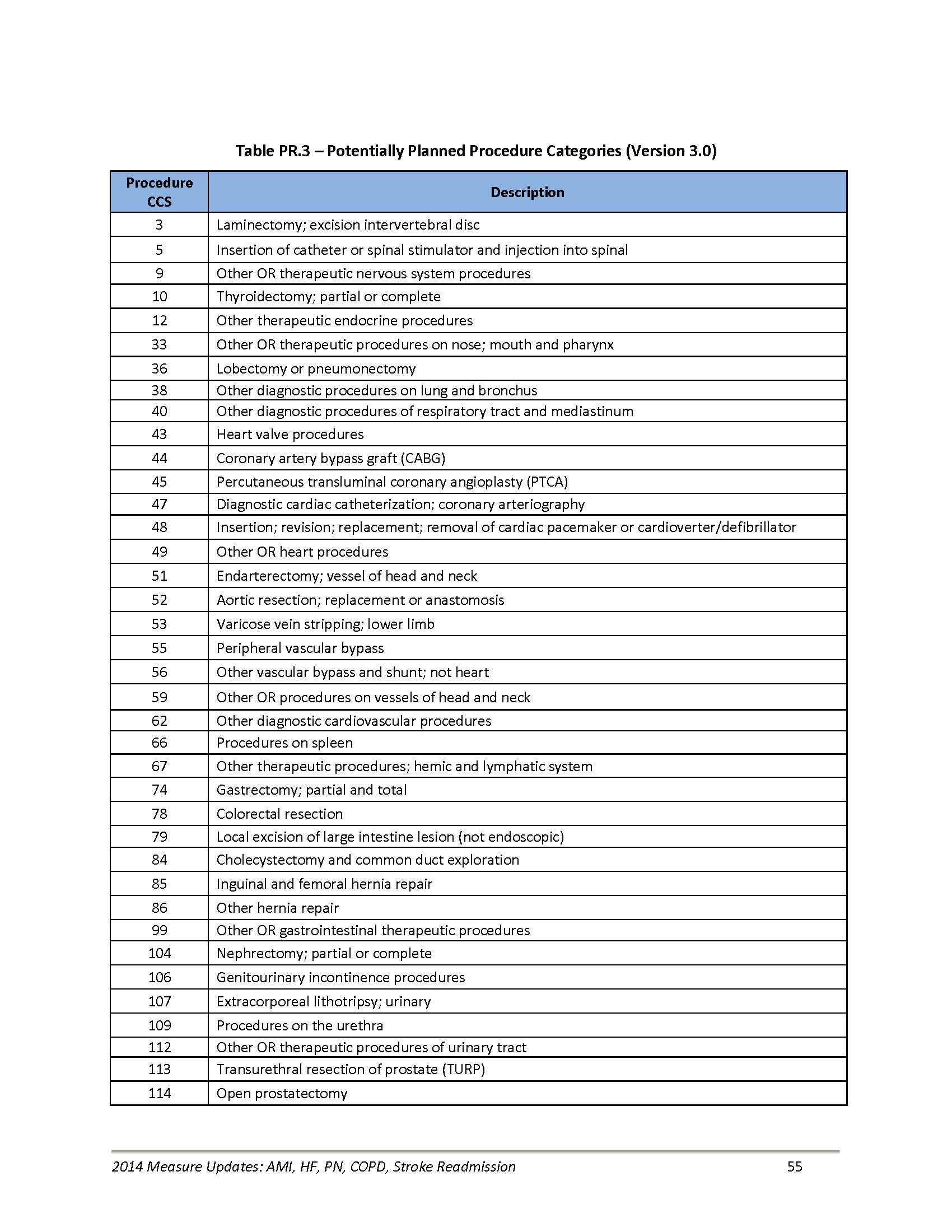
Appendix Exhibit 3, Continued**

**
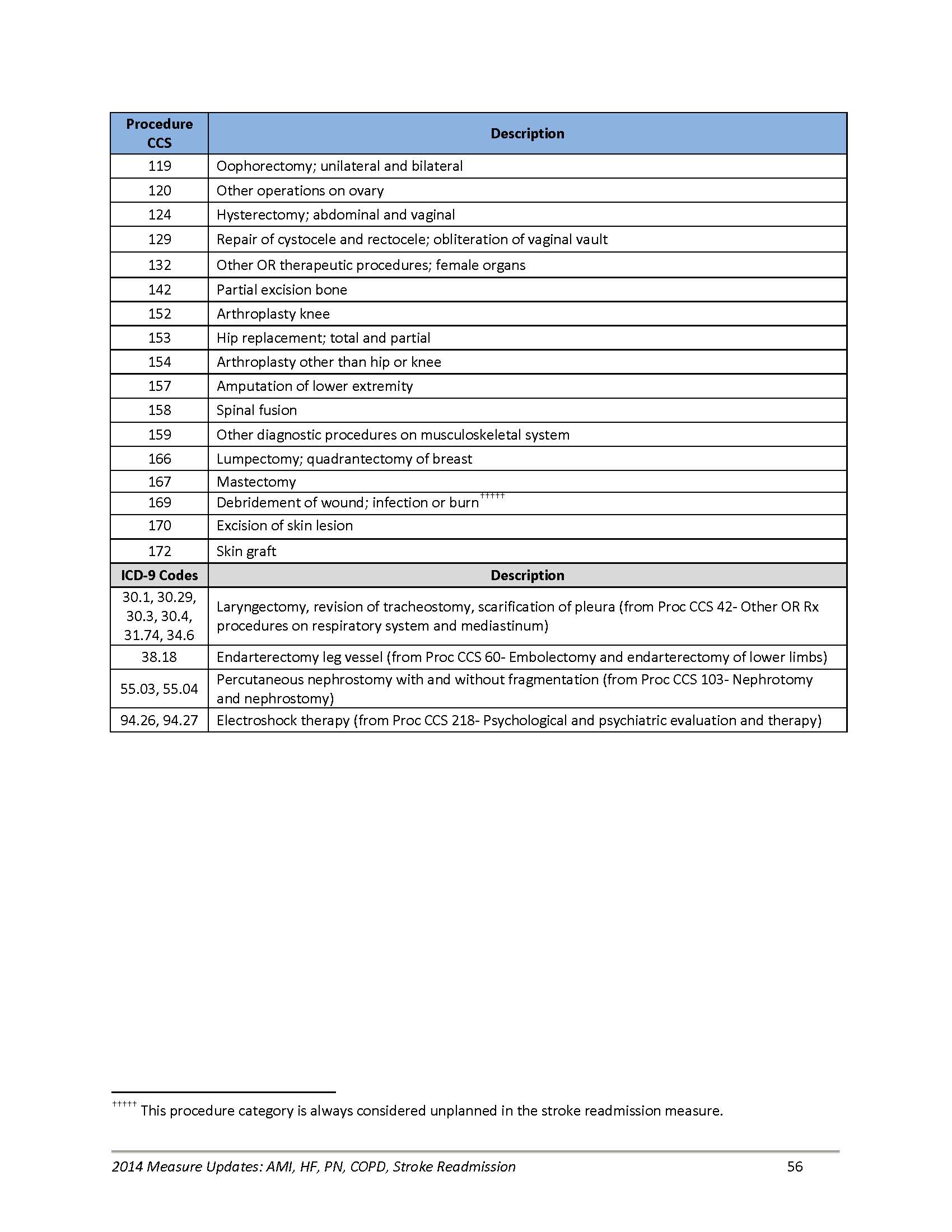
**

**Appendix Exhibit 3, Continued**

**
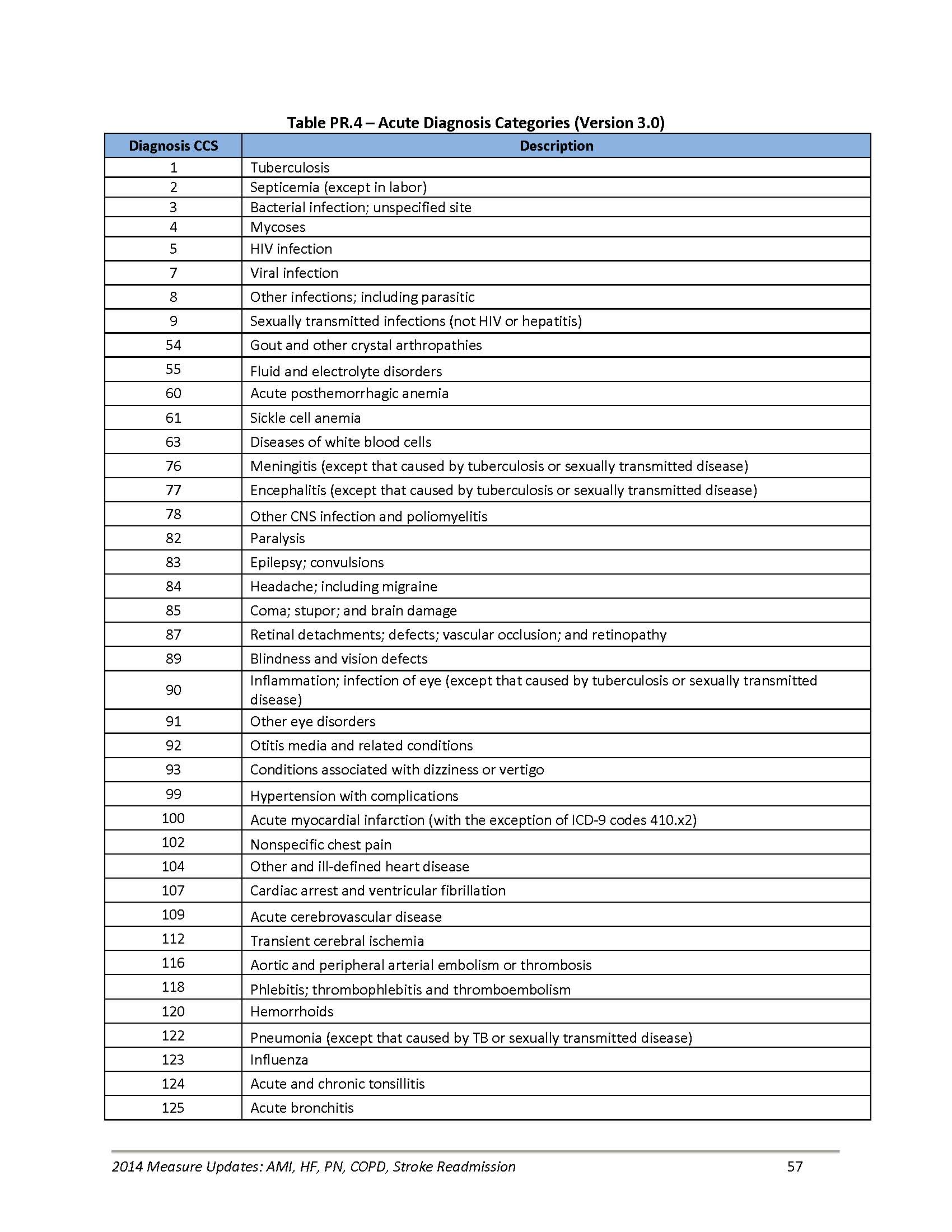
**

**Appendix Exhibit 3, Continued
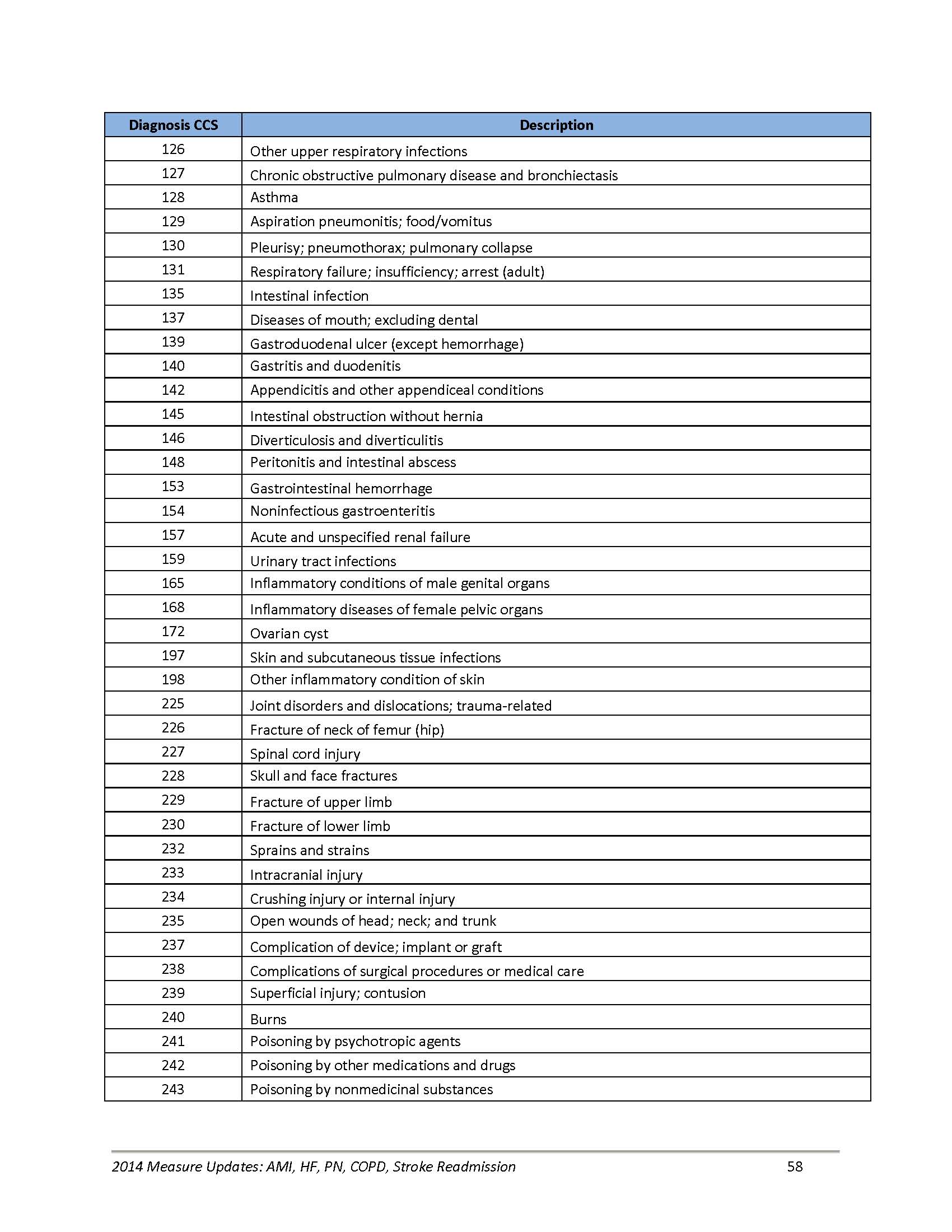
**

**Appendix Exhibit 3, Continued**

**
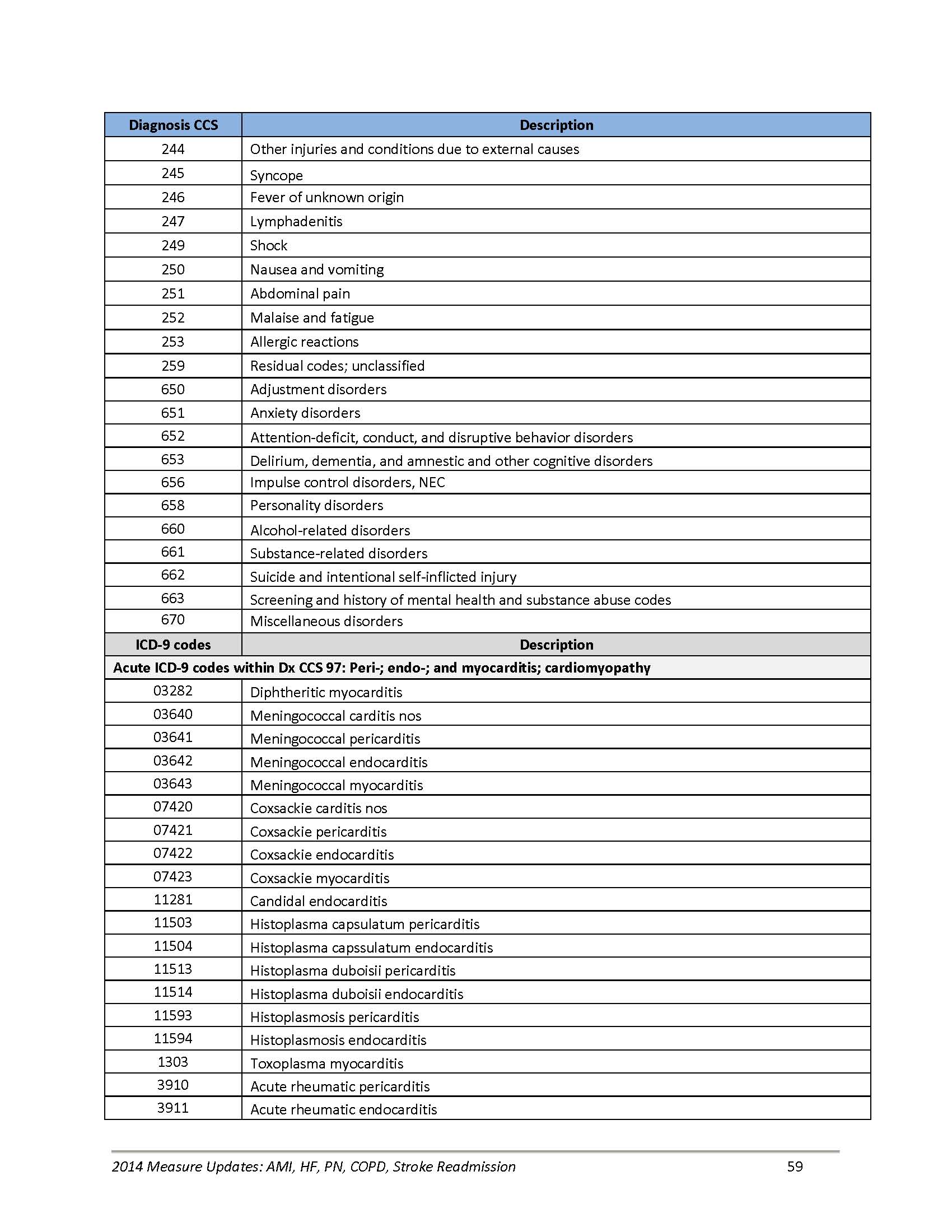
**

**
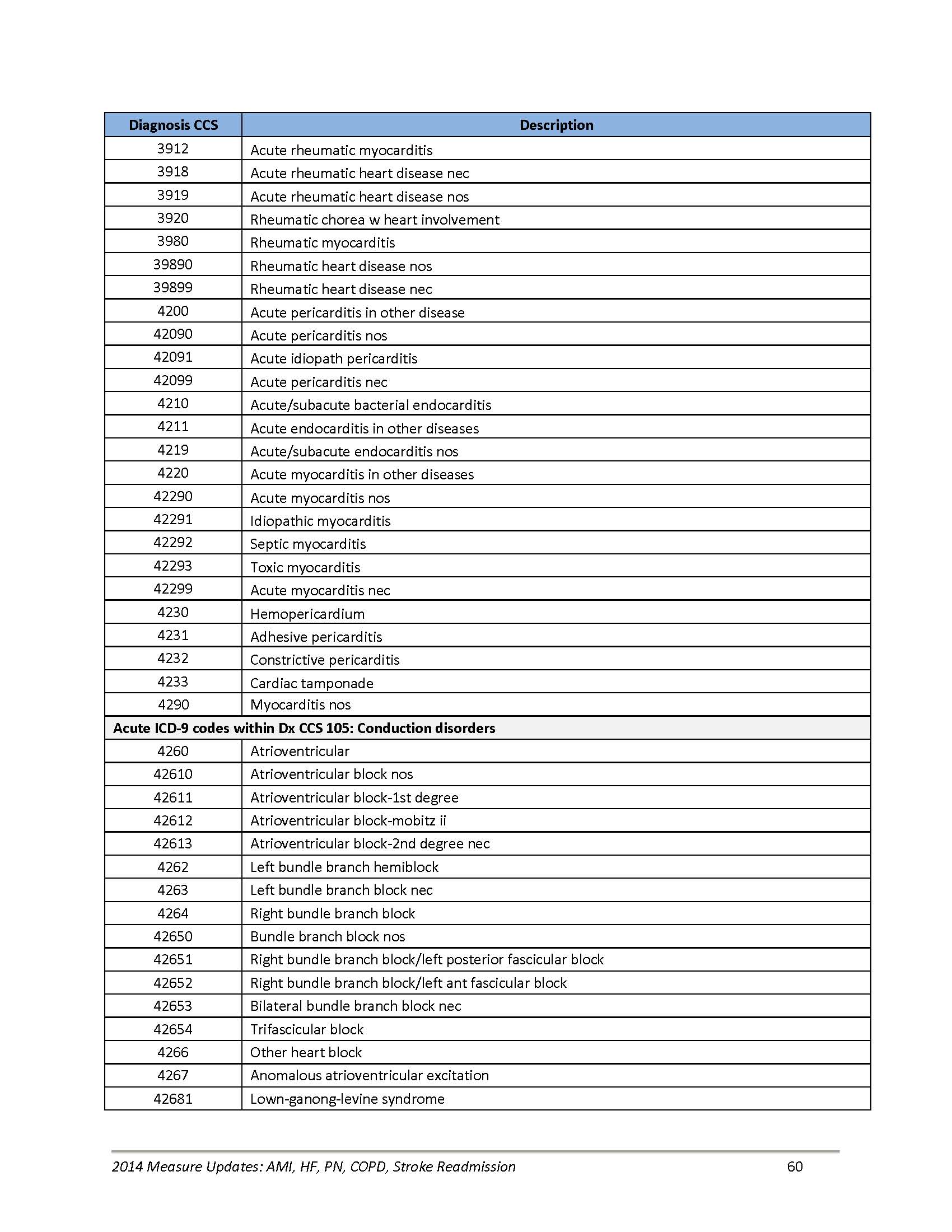
Appendix Exhibit 3, Continued**

**Appendix Exhibit 3, Continued**

**
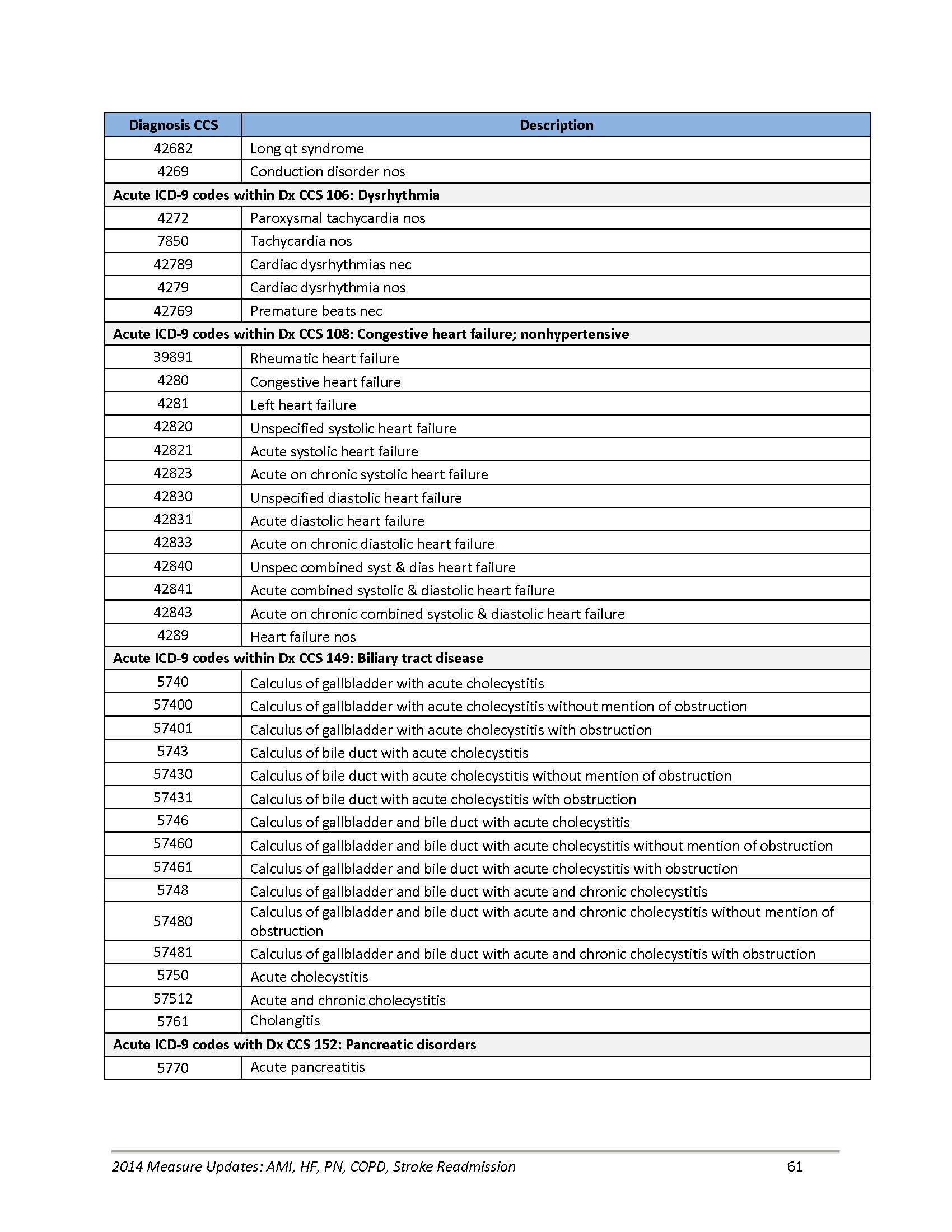
**

**Appendix Exhibit 4: Risk Adjustment Coefficients for**

**AHRQ Inpatient Quality Indicator #15**

**
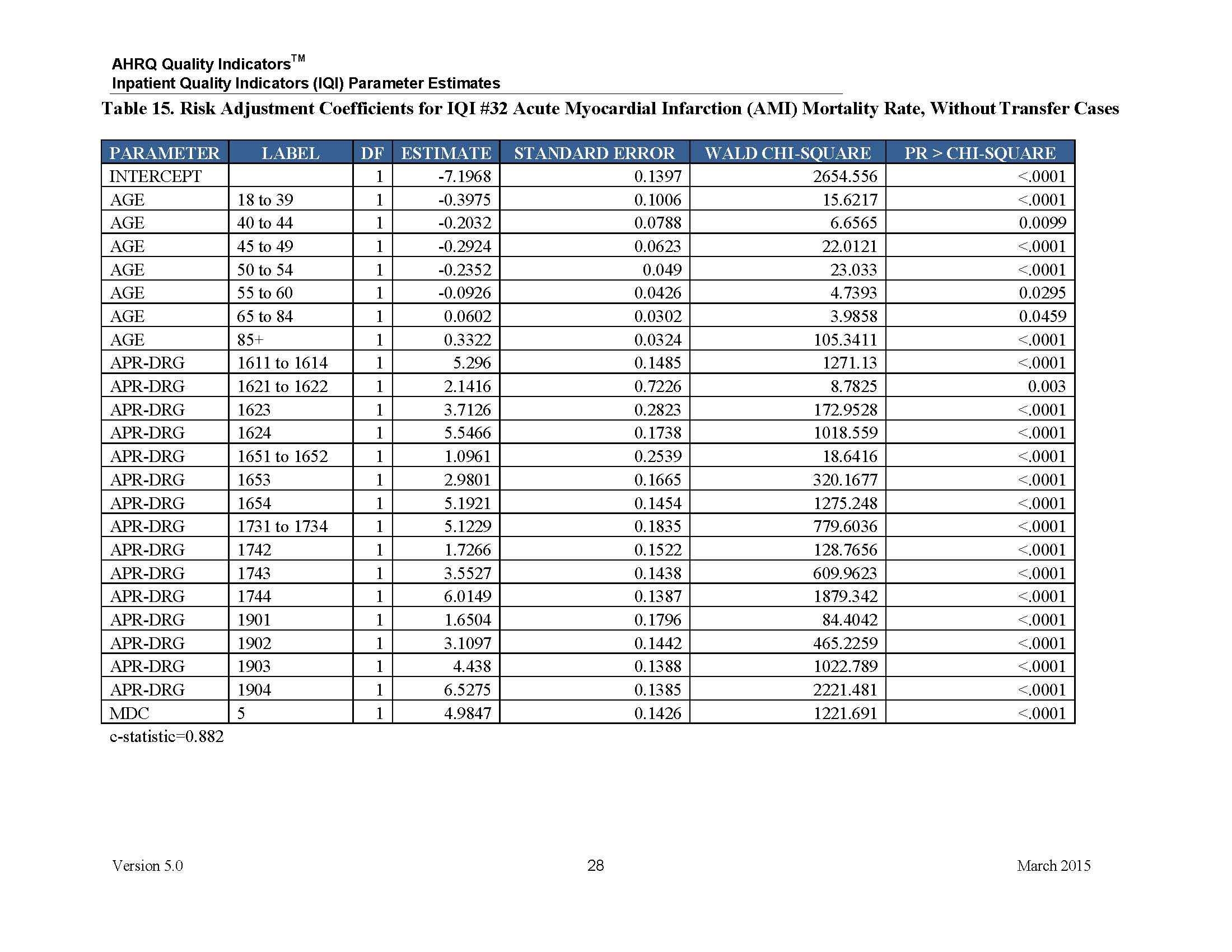
**

**Appendix Exhibit 5: Summary Statistics for Analysis Sample**

**Appendix Exhibit 6: Estimation Results from Primary Analysis**

1. Specifically, for advanced cardiac care, the DRGs were 1, 2, 216-221, and 231-236. For advanced neurologic care, the codes were 25-27 and 955. [↑](#footnote-ref-2)
